# A review of post-GWAS prioritization approaches

**DOI:** 10.3389/fgene.2013.00280

**Published:** 2013-12-09

**Authors:** Lin Hou, Hongyu Zhao

**Affiliations:** Department of Biostatistics, Yale School of Public HealthNew Haven, CT, USA

**Keywords:** genome-wide association studies, prioritization, eQTL, DNase I hypersensitive site, non-coding

## Abstract

In the recent decade, high-throughput genotyping and next-generation sequencing platforms have enabled genome-wide association studies (GWAS) of many complex human diseases. These studies have discovered many disease susceptible loci, and unveiled unexpected disease mechanisms. Despite these successes, these identified variants only explain a small proportion of the genetic contributions to these diseases and many more remain to be found. This is largely due to the small effect sizes of most disease-associated variants and limited sample size. As a result, it is critical to leverage other information to more effectively prioritize GWAS signals to increase replication rates and better understand disease mechanisms. In this review, we introduce the biological/genomic features that have been found to be informative for post-GWAS prioritization, and discuss available tools to utilize these features for prioritization

## INTRODUCTION

With the developments of affordable and reliable high-throughput genotyping and next-generation sequencing platforms, many genome-wide association studies (GWAS) have been successfully conducted to identify DNA variants associated with many complex human diseases and traits, such as cancer, autoimmune diseases, height, blood pressure, body mass index, among others. As of 11/16/13, there were 11,907 single nucleotide polymorphisms (SNPs), 940 traits with 15,052 associations documented in the GWAS catalog, maintained by the National Human Genome Research Institute (Hindorff et al., 2009). These studies have uncovered many novel genes and implicated unexpected pathways associated with disease mechanisms, leading to great insights on disease etiology.

In spite of these accomplishments, many challenges remain in GWAS design and analysis. The first limitation is the limited statistical power to identify all disease-associated loci. Although many susceptible loci have been identified, they only explain a small fraction of the overall heritability, with the majority of heritability remaining unexplained. One possible reason is that the missing heritability is due to the lack of coverage of genetic variations on the genotyping platforms, such as those rare or even private variations. Another explanation is that most disease-associated variants have small effect sizes that are not likely detected due to low statistical power, even with thousands of subjects. To better identify these variants, more powerful and cost-effective designs and statistical methods are desired. Several approaches have proved cost-effective to enrich signals and increase statistical power. For example, a number of customized genotyping platforms have been used to target certain genomic regions with high density to fine map disease-associated variants. The successful examples include the use of the ImmunoChip (Trynka et al., 2011) to fine map 186 distinct loci associated with 12 autoimmune diseases, and the use of the MetaboChip (Voight et al., 2012) to fine map established trait-associated loci. As for rare variant analysis, a number of whole exome sequencing studies have enjoyed success for diseases like autism and schizophrenia (Xu et al., 2011; Sanders et al., 2012). It has also been found that studies focusing individuals with extreme phenotypes can increase statistical power because of the enriched genetic signals in the study subjects (Lin et al., 2013). In addition to improved platforms and study designs, many statistical methods have also been developed to increase statistical power. Meta-analysis is commonly applied to leverage all the information from separate studies to increase statistical power to identify disease-associated loci. A number of methods aim to investigate the combinatorial effects of a group of SNPs, including both marginal and interaction effects. These are accomplished by explicitly modeling the interactions of two or more SNPs; joint analysis of all the SNPs in a gene or defined region; and joint consideration of the SNPs in proximity of all the genes annotated in a specific pathway/network. The advances in study designs and statistical methods have led to many novel discoveries. For example, a recent study of the inflammatory bowel disease (IBD; Jostins et al., 2012), which is a meta-analysis of both ImmunoChip and GWAS datasets, increased the number of IBD loci to 163, where these loci account for 13.6% of the genetic risk of Crohn’s disease (CD) and 7.5% of ulcerative colitis. See Cantor et al. (2010) for a comprehensive review of some of the topics.

The second limitation is the difficulty to interpret the biological relevance of susceptible loci and link them with the disease etiology. Because the ultimate goal of association studies is to understand disease etiology and develop effective strategies to prevent and treat diseases, it would be desirable that GWAS results can implicate functional variants and disease pathways that can be experimentally studied in follow-up functional studies. However, a large proportion of disease-associated variants fall into non-coding regions of the genome, with 88% of the associated SNPs in GWAS catalog non-coding (Hindorff et al., 2009), making it difficult to form testable hypothesis. Even when the variants in the coding region, it is often not clear whether they are functional due to the presence of several closely linked variants. To address this problem, many statistical methods have been proposed to prioritize GWAS signals by incorporating diverse functional evidence, so that variants with small effect sizes but possessing functional features may be prioritized over variants with similar effect sizes but less likely to be functional. GWAS signals can be prioritized at both the SNP level and gene level, depending on the biological features considered and the input signals. Statistical approaches that prioritize at the SNP level are especially helpful in pinpointing the causal variants with sequence data, where essentially all the variations in the genome can be identified. This is contrast to earlier GWAS that only interrogated a subset of SNPs, such as tag SNPs, in genotyping platforms. One benefit of such approaches is that the functional evidence provides paths to derive plausible and testable hypotheses for the prioritized genes or loci. Moreover, with the incorporation of other data informative about disease association, the prioritized genes/loci are more likely to be truly associated with disease. For example, it has been observed that trait-associated loci are more concentrated in regions with certain genomic features, such as protein coding regions and expression quantitative trait loci (eQTL). In this review, we will review (1) biological/genomic features that are informative for prioritizing GWAS results; and (2) computational methods and tools that prioritize disease-associated SNPs by integrating these biological/genomic features.

## BIOLOGICAL FEATURES USED IN PRIORITIZATION AND THEIR JUSTIFICATIONS

The first step, and sometimes the only step of many SNP prioritization approaches is to annotate the candidate SNPs by intersecting GWAS signals with desired genomic features, such as eQTLs, transcription factor binding sites, DNase hypersensitive sites, histone modifications, and others. For CD, Fransen et al. (2010) showed that *cis*-eQTL SNPs were enriched in known CD-associated SNPs. Based on this observation, the authors proposed to select a subset of SNPs to follow-up a public CD GWAS dataset, to intersect the top 500 GWAS hits with *cis*-eQTLs in an eQTL database. The SNPs thus selected, *cis*-eQTLs of the genes *UBE2L3* and *BCL3*, were replicable in two independent replication cohorts. This represents a successful application of the annotation-based prioritization methods.

The genomic features may implicate functional roles of the prioritized SNPs to disease etiology, and these hypotheses can be formally tested through molecular studies. These include variants both in coding and non-coding regions. Through these filtering/intersecting methods, researchers can focus on a much smaller number of SNPs in follow-up studies. Although the proximity of a SNP with a documented genomic feature may suggest a functional role of the SNP, it may not necessarily increase the probability that it will affect the phenotype of interest, nor the probability that the locus is truly susceptible. In general, the genomic features discussed above are considered biologically plausible and extensively used to prioritize SNPs, but whether each feature is informative on a SNP’s functional relevance is disease and context dependent. In a recent study, Minelli et al. (2013) tried to identify features that are important in selecting SNPs for follow-up studies by surveys in experts. They sent questionnaires to ten experts who conducted GWAS studies, and asked their opinion on the importance of a set of selected features. The features included relative position of the SNP to a nearby transcript, whether the SNP causes an amino acid change, etc. (see Table 2 in their paper). The result was not surprising, as experts considered gene level evidence more important, such as the SNP in a gene that is previously associated with the phenotype, or that encodes a protein in a phenotype related pathway, or that has gene/protein interaction relevant to the phenotype. Experts opinions are valuable, however, they might be biased toward existing knowledge and also expertise in specific diseases. Nevertheless, this paper highlighted the need for understanding what features should be considered in prioritization. In the following, we review these features and statistical methods to use these features, to inform human geneticists in their applications of the annotation-based approaches.

### EXPRESSION QUANTITATIVE TRAIT LOCI

By contrasting the SNPs documented in the GWAS catalog (Hindorff et al., 2009) with those randomly sampled SNPs with matching minor allele frequency distribution, Nicolae et al. (2010) showed that complex trait-associated SNPs are more likely to be eQTLs. The conclusion remained valid for a linkage disequilibrium (LD)-pruned subset of SNPs in the GWAS catalog. Since the eQTL annotation considered by Nicolae et al. (2010) was derived from an expression dataset of lymphoblastoid cell lines, it was of interest to investigate whether cell line-specific eQTLs showed different levels of enrichments across diseases with different focal tissues, including cancer, neurological/psychiatric disorders, and autoimmune disorders. By tissue of relevance, the lymphoblastoid cell lines should be a good proxy for autoimmune disorders, and relatively poor for cancer and neurological/psychiatric disorders. As expected, there was greater enrichment of eQTLs in the group of autoimmune disorders, while only moderate enrichment for the other two groups of diseases. Furthermore, in the examination of the results in the Wellcome Trust Case Control Consortium (WTCCC) GWAS dataset of CD, eQTLs were enriched in SNPs with association *p*-value less than 0.01, but not for the missense SNPs, indicating potential loss of information if non-coding SNPs are ignored.

### PROTEIN DELETERIOUSNESS PREDICTIONS

Polymorphisms in the coding region may have different effects on protein function. Synonymous SNPs do not change the corresponding protein sequence; non-synonymous SNPs change amino acid composition, or truncate the protein sequence by causing an early codon; indels can change protein sequence with varying consequence depending on whether the indel is in-frame or frame-shifting; SNPs and indels can also interrupt splicing sites, thus change the mRNA isoform. In other words, mutations in the coding region may be benign or deleterious to protein function, with deleterious mutations more likely to have a phenotypic effect. Many computational tools have been developed to predict “deleteriousness” of SNPs and indels (Ng and Henikoff, 2003; Adzhubei et al., 2010). These methods generally take features like biochemical property of the altered amino acid, conservation and sequence homology as input, and use machine learning technique to train a classifier. These methods have been comprehensively reviewed by Cooper and Shendure (2011) and Ng and Henikoff (2006).

The most extreme case of protein function interruption is the loss of function mutations. However, genome sequencing studies found that all human carry loss of function mutations without obvious phenotypic effect, and common loss of function variants were depleted in polymorphisms associated with complex disease like CD and rheumatoid arthritis (MacArthur et al., 2012). The results indicate that the “deleteriousness” feature should be interpreted with caution, since disruption of protein function does not necessarily have a phenotypic effect. In this regard, the “residual variance intolerance score” has been defined quantitatively measure the tolerance of a protein to mutations (Petrovski et al., 2013). The number of missense and non-sense variants found in each gene in the cohort of the National Heart, Lung, and Blood Institute (NHLBI) exome sequencing project was compared to the number of functionally neutral variants, and genes with variants fewer than expected are assigned a negative score, indicating they are less tolerant to variations.

### DIFFERENTIAL GENE EXPRESSIONS

Gene expression microarrays and RNA-seq are commonly used to study gene expression profiles in disease cases and matched controls, and differentially expressed genes thus identified may suggest disease mechanisms and potential biomarkers that can be further explored in follow-up studies. Chen et al. (2008) analyzed 476 expression datasets in the Gene Expression Omnibus (GEO), and calculated the frequency that a gene was differentially expressed in these datasets, which they called “differential expression ratio.” They found that differential expression ratio is positively correlated with the likelihood that a gene harbors disease-associated variants, where the list of disease-associated genes was created by combining information from Genetic Association Database (GAD; Becker et al., 2004) and Human Gene Mutation Database (HGMD; Stenson et al., 2003). In addition, they found that among the genes discovered in the initial scan of the WTCCC type 1 diabetes mellitus GWAS dataset, the differential expression ratio was higher in genes that were replicable than those not replicable in follow-up studies. These authors have developed an online server, FitSNPs, to incorporate this feature (see **Table 1**).

**Table 1:** A list of online SNP prioritization tools.

Name	Website	Reference
FASTSNP		Yuan etal. (2006)
FitSNPs		Chen etal. (2008)
SNPranker 2.0		Merelli etal. (2013)
SPOT		Saccone etal. (2010)

### DNase I HYPERSENSITIVE SITES

DNase I hypersensitive sites (DHSs) are markers of accessible chromatin, which indicate regulatory roles in the transcription process. DHS have been mapped in 349 cell and tissue samples genome-wide by next-generation sequencing (Thurman et al., 2012). Enrichment analysis showed that trait-associated SNPs in the GWAS catalog (Hindorff et al., 2009) are more concentrated within DHS regions, excluding confounding factors such as allele frequency and distance from the nearest transcriptional start site (Maurano et al., 2012).

### OTHERS

There are many more genomic features collected and annotated in large community projects, such as the Encyclopedia of DNA Elements (ENCODE; Consortium, 2011), which are potentially valuable for SNP prioritization. Kindt et al. (2013) examined enrichment or depletion of trait-associated SNPs in 58 genomic features. The features investigated covered genic and regulatory features, conservation features, and chromatin state features (see Table 1 in Kindt et al., 2013). Among those features, genomic regions annotated as “heterochromatin” and “low expression signals” are depleted of trait-associated SNPs, while eQTLs and “strong enhancer” showed the highest level of enrichment.

The biological features discussed so far are measured/inferred from laboratory cell lines and the sequence and annotation of the human genome, which do not provide trait-specific information. However, trait-relevant features are intrinsically helpful for prioritization. For example, a DNase-seq experiment in intestine tissues and immune cells of CD patients would be more informative for prioritizing variants associated with CD than those measured in brain tissues. Maunakea et al. (2010) and Portela and Esteller (2010) reviewed recent progress in mapping the epigenome (including DNA methylation and histone modification), showing that epigenetic modifications play important roles in human diseases, including cancer, neurodevelopmental disorder, neurodegenerative disease, neurological disease, and autoimmune diseases. Thus, epigenome data in disease states is valuable for understanding disease and prioritize disease susceptible loci. However, efforts in disease-specific epigenome mapping and systematic database to document such data are still lacking and greatly needed. For DNA methylation alone, a database, DiseaseMeth, has incorporated methylation data for 72 human diseases (Lv et al., 2012).

## SNP PRIORITIZATION APPROACHES AND AVAILABLE WEB SERVERS

Here we review methods and tools that prioritize GWAS signals at single SNP level. There are mainly two steps in these methods. The first step applies annotations or filters based on whether or not the candidate SNP has the desirable features and the second step scores the candidate SNPs by integrating evidence from multiple sources. Saccone et al. (2008, 2010) developed an online prioritization tool, SPOT, which systematically combines multiple biological databases to prioritize SNPs by the genomic information network (GIN) model (see **Table 1**). In their model, each SNP is assigned a prioritization score, which is a linear sum of scores derived from pathway information, comparative genomics, linkage scan, and results of other independent GWAS studies. The weights are decided by the strength of the link between the SNP and the annotations. For example, for a SNP that is in LD with a non-synonymous SNP of a susceptible gene, the assigned weight will be less than that of SNPs physically in the gene. The methodology prioritized SNPs with increased biological relevance in a GWAS study of nicotine dependence. Thompson et al. (2013) incorporated biological features in a Bayesian framework, where the prior probability that a SNP is associated with the phenotype is determined by its annotations. They first curated a training set, including SNPs that were confirmed replicable as the positive set, and 1,000 randomly selected SNPs as control set. For a selected set of features, a logistic regression model was fit for each disease. Thus, the log odds ratio that a test SNP is associated with the disease can be estimated through the model.

There are also web servers that perform SNP prioritization in an annotation fashion. They annotate the candidate SNPs by single or multiple features, but do not combine the results. They differ by the features and strategies they use in prioritization. A list of SNP prioritization resources are provided in **Table 1**. Most of these tools are only applicable to SNPs, and tools that can prioritize indels are still lacking.

*FASTSNP* uses a decision tree framework to assign different risk level to SNPs by considering the genomic location and functional effect of the SNPs (Yuan et al., 2006).

*FitSNPs* calculated a differential expression ratio for all genes in the genome, and prioritize SNPs by the differential expression ratio of their associated genes (Chen et al., 2008).

*SNPranker 2.0* first annotates the SNPs with different features, and then user a user interactive way to integrate features (Chen et al., 2008). Users can specify the features they want to include and the weight of each feature, which would give the users an opportunity to enforce their biological priors. But they also provide an optimal set of weights by default. The optimal weights were determined by a cross-validation approach.

## TOOLS FOR VARIANT ANNOTATION

Besides the SNP prioritization tools, there are also many web servers and software for variant annotation (**Table 2**), which could provide useful information for prioritization. Basically, these tools take a list of query variants as input, and annotate them with their in-house databases. Among these, HaploReg (Ward and Kellis, 2012a) and RegulomeDB (Boyle et al., 2012) provide annotation for variations in non-coding regions. HaploReg annotates variations by their chromatin state, conservation across mammals, and computationally predicted transcription factor binding sites. Besides, by utilizing LD information from the 1000 Genomes Project, HaploReg automatically reports, and annotates all variations within a user-specified LD threshold of the query variant. RegulomeDB has incorporated many data sources, including the ENCODE project, available transcription factor ChIP-seq data, and eQTL datasets. The other tools are designed for variations in the whole genome. They annotate the query variations by dbSNP ID, allele frequency in different ethnic groups, position in a transcript (intron, exon, 5′ UTR, etc.,), and the resultant amino acid change if any. SeattleSeq (Ng et al., 2009) and Variant Effect Predictor (VEP; McLaren et al., 2010) has convenient web interface, suitable for users who are not familiar with scripts and programming languages, while ANNOVAR (Wang et al., 2010) and Snpeff (Cingolani et al., 2012) are stand-alone software packages, so that they can be easily incorporated into variant analysis pipelines. Discussions on variant annotation tools can also be found in Ward and Kellis (2012b).

**Table 2:** list of tools for variant annotation.

Name	Website	Reference
ANNOVAR		Wang etal. (2010)
HaploReg		Ward and Kellis (2012a)
RegulomeDB		Boyle etal. (2012)
SeattleSeq		Ng etal. (2009)
Snpeff		Cingolani etal. (2012)
VEP		McLaren etal. (2010)

## DISCUSSION

In this review, we have focused on biological and genomic features that are informative for SNP prioritization. In the second phase of association studies, researchers can use these databases or tools to choose SNPs in follow-up studies. The observation that eQTLs and open chromatin regions are enriched of trait-associated SNPs highlights the potential rich information in the non-coding regions of the genome (Nicolae et al., 2010). Nonetheless, the gene-centric approaches may be helpful in disease gene discovery, and there are many approaches that perform prioritization at the gene level. A review of the methods and tools for gene-based prioritization can be found in Tranchevent et al. (2011).

The prioritization methods discussed here take as input a list of candidate SNPs, which is usually derived by taking all the SNPs achieving a specified significance level in GWAS, e.g., all SNP with *p*-values less than 0.01. The SNPs are treated equally regardless of the association *p*-values. However, the *p*-value, the effect size, and other statistics that summarize the association level of individual SNP could be informative for SNP selection. A computational framework that incorporates the significance level with the biological/genomic features discussed above might improve the performance of the prioritization scheme. A discussion of different signal measures of association was given by Strömberg et al. (2009).

Although there are many web servers and databases for SNP prioritization, most of them provide annotations of different types of features, but do not rank these SNPs through integrating GWAS and annotation information. Also, these methods do not employ disease-specific information. There is still a great need for statistical methods that select and integrate multiple annotations in a disease-specific manner, and re-rank SNPs under a coherent statistical framework.

## Conflict of Interest Statement

The authors declare that the research was conducted in the absence of any commercial or financial relationships that could be construed as a potential conflict of interest.

## Author Contributions

Lin Hou and Hongyu Zhao conceived and wrote the paper.
